# An investigation of qualitative variations of groundwater resources under municipal wastewater recharge using numerical and laboratory models, Nazarabad plain, Iran

**DOI:** 10.1007/s11356-021-12638-x

**Published:** 2021-06-18

**Authors:** Nezhla Amiri, Mohammad Nakhaei

**Affiliations:** Department of Applied Geology, Faculty of Earth Sciences, Kharazmi University, Mofateh Ave, Tehran, Iran

**Keywords:** Treated municipal wastewater, Soil column experiment, Contaminant transport modeling, HYDRUS-1D, Trace element, Heavy metal, Nazarabad plain

## Abstract

Municipal wastewater irrigation induces elevated concentrations of heavy metals in the soil which their further leaching leads to groundwater contamination in the long run. In this study, both column experiment and 5-year prediction modeling using HYDRUS-1D were conducted to investigate the probable adsorption and transport of 10 different metals including As, Ba, Cr, Cu, Mo, Ni, Pb, Rb, Sr, and Zn in an alkaline soil from Nazarabad plain in Iran which has been irrigated with treated urban wastewater for several years. The obtained results revealed that reaching the equilibrium rate for the mentioned elements during 1825 days (= 5 years) was as follows: Mo > Cr > Rb > Zn > Ni > Ba> Sr > Pb > As> Cu. The finding implies that molybdenum (Mo) and copper (Cu) are the most mobile and the most adsorbent heavy metals in the soil, respectively. Higher mobility poses the greater potential risk of leaching into groundwater resources. Overall, experimental and numerical modelings had good accordance and were capable of describing the actual condition.

## Introduction

Municipal wastewater is an effluent that is produced mainly by domestic and non-industrial commercial activities (Maliva and Missimer 2012). While suspended and soluble organic and inorganic solids have a little share, 99.94% of its total volume consists of pure water (Abel 2014; Drinan and Spellman 2013; Pescod 1992). Compared to other types of effluents, municipal wastewater reuse is more possible due to its characteristics (Charkhestani et al. 2015). Re-consumption of wastewater has an old history all over the world, and the indications of its reuse date back to at least 5000 years ago and more precisely to the Minoan civilization of ancient Greece (Maliva and Missimer 2012). Nowadays, the major use of wastewater is in irrigation, which is the direct/indirect non-potable reuse of wastewater (Fatta-Kassinos and Kummerer 2016; Keremane 2017). Providing requisite water in arid regions, supplying required plant nutrients and thus reducing the expenses concerning agronomic fertilizers, and ultimately being a secondary water resource increasing in amount along with population growth are some benefits of wastewater reuse (Jafari et al. 2018; Maliva and Missimer 2012).

From another aspect, reutilizing wastewater for irrigation due to exceeding the maximum admissible concentrations for contaminants has some negative effects on corps, soil, and groundwater (Aydin et al. 2015; Belhaj et al. 2015). While numerous papers have been conducted to understand the impact of wastewater on groundwater resources (Alslaibi et al. 2017; Gotkowitz et al. 2016; Martins et al. 2017; Missimer et al. 2012; Siebe et al. 2018), many researchers have performed soil column experiments and HYDRUS-1D modeling to understand the impact of exclusively municipal wastewater on basic soil properties, absorption or movement of trace elements in soil, and the further possibility of their recharge into groundwater resources which causes pollution in the long term (Mahmoudi et al. 2018; Mojid et al. 2019; Mamindy-Pajany et al. 2013).

Moreover, monitoring the mobility of trace elements and especially heavy metals (HM) in the soil is important because once they release into the soil-subsurface environment, they cannot be degraded (Berkowitz et al. 2014). If their levels are above the defined standard limits and can reach groundwater through leaching, it can endanger public health, because from the global standpoint, nearly 1.5 billion people depend upon groundwater for their drinking water supply and, in the absence of drinking water, people are bound to use pond water which hastens various types of waterborne diseases. Approximately, one-sixth of the world’s population does not have access to clean drinking water which makes us pay more attention to this issue (Eslamian and Eslamian 2017). For instance, the presence of arsenic in groundwater poses the greatest health risk to humans due to the direct ingestion of arsenic-contaminated well waters, compared to its presence in rainwater or surface waters. This is all the more important given the fact that arsenic does not alter any of the physical properties of water (taste, color, and odor); it takes years for the symptoms of the related disease to appear and in most cases is fatal (Ahuja 2008). Also, about 300 million people worldwide are exposed to arsenic contamination in their drinking water and food supplies (Fares and Singh 2020). As a result, not only arsenic, as an example, but also the behavior and mobility of other trace elements in soil should be studied.

Nazarabad city with the area of 576 *km*^2^ and 150,000 population is located 85 km west of Tehran, inside the central zone of Iran. In this region, average annual rainfall, temperature, and humidity are 229.4 mm, 2.14 °C, and 45%, respectively. The climate of the region is also semi-arid. An examination of the soil of Nazarabad plain shows that it is chiefly coarse and fine-grained sediments that have been washed from the high northern areas during different geological eras and raining cycles and have been deposited subsequently in the southern regions. Moving from north to south, most sediments turn from coarse grained to fine grained, and the soil is very suitable for agriculture (especially irrigation farming) in terms of material and depth. The main water resources in Nazarabad are supplied by groundwater, which in the past was used in the form of manual wells and aqueducts (qanats) and in recent years in the form of deep and semi-deep wells. Additionally, the common type of aquifer in this area is often semi-permeable.

The aim of this study was to simulate the movement and transfer of trace elements in soil by creating a soil column experiment and numerical modeling, identifying soil self-purification, and examining the possibility of using treated municipal wastewater in irrigation without adversely affecting groundwater resources.

## Materials and methods

### Soil sampling

**Fig. 1:**
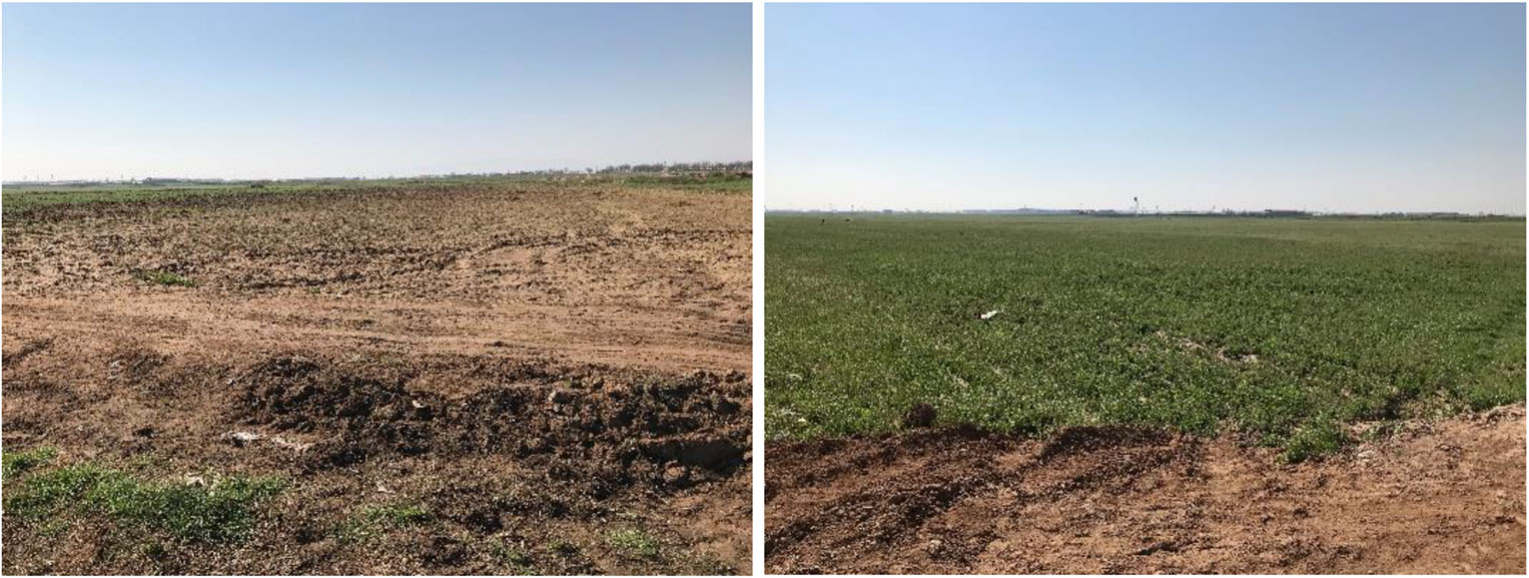
Agricultural lands that have been irrigated or flooded with the treated wastewater

Soil sampling was carried out in April 2019. The soil samples were randomly taken from 12 different points at a depth of 40 to 60 cm from agricultural lands that have been irrigated or flooded with the treated wastewater (Fig. 1). Then, to obtain one representative soil sample, all the 12 soil samples were mixed together. In the following, the mixed soil was air-dried, sieved (less than 2 mm according to ASTM D6913 standard (ASTM-International 2014)), and homogenized. Table 1 shows the physico-chemical characteristics of the representative soil sample. Also, the texture of the representative soil sample was sandy based on the Udden-Wentworth grade scale for grain size (Douglas and McConchie 1994). X-ray diffraction (XRD) analysis detected quartz (*SiO*_2_), calcite (*CaCO*_3_), and albite (*NaAlSi*_3_*O*_8_) as existing major minerals in the representative soil sample. Illite ((*K*, *H*_3_*O*)*Al*_2_*Si*_3_*AlO*_10_(*OH*)_2_), orthoclase (*KAlSi*_3_*O*_8_), chlorite ((*Mg*, *Fe*)_6_(*Si*, *Al*)_4_*O*_10_(*OH*)_8_), and dolomite (*CaMg*(*CO*_3_)_2_) were available as minor minerals in the so-called sample.

**Table 1:** Physico-chemical characteristics of the representative soil sample

Parameter	Amount	
Porosity (%)	38	
Bulk density (*g*/*cm*^3^)	1.81	
pH	8.31	
EC (μS/cm)	472	
Effective size (*D*_10_) (mm)	0.11	
Sand (%)	Very coarse sand (%13.08)	97.57
	Coarse sand (%16.97)	
	Medium sand (%20.63)	
	Fine sand (%34.38)	
	Very fine sand (%12.18)	
Silt and clay (%)	2.43	

### Wastewater sampling and characteristics of treated wastewater

**Fig. 2:**
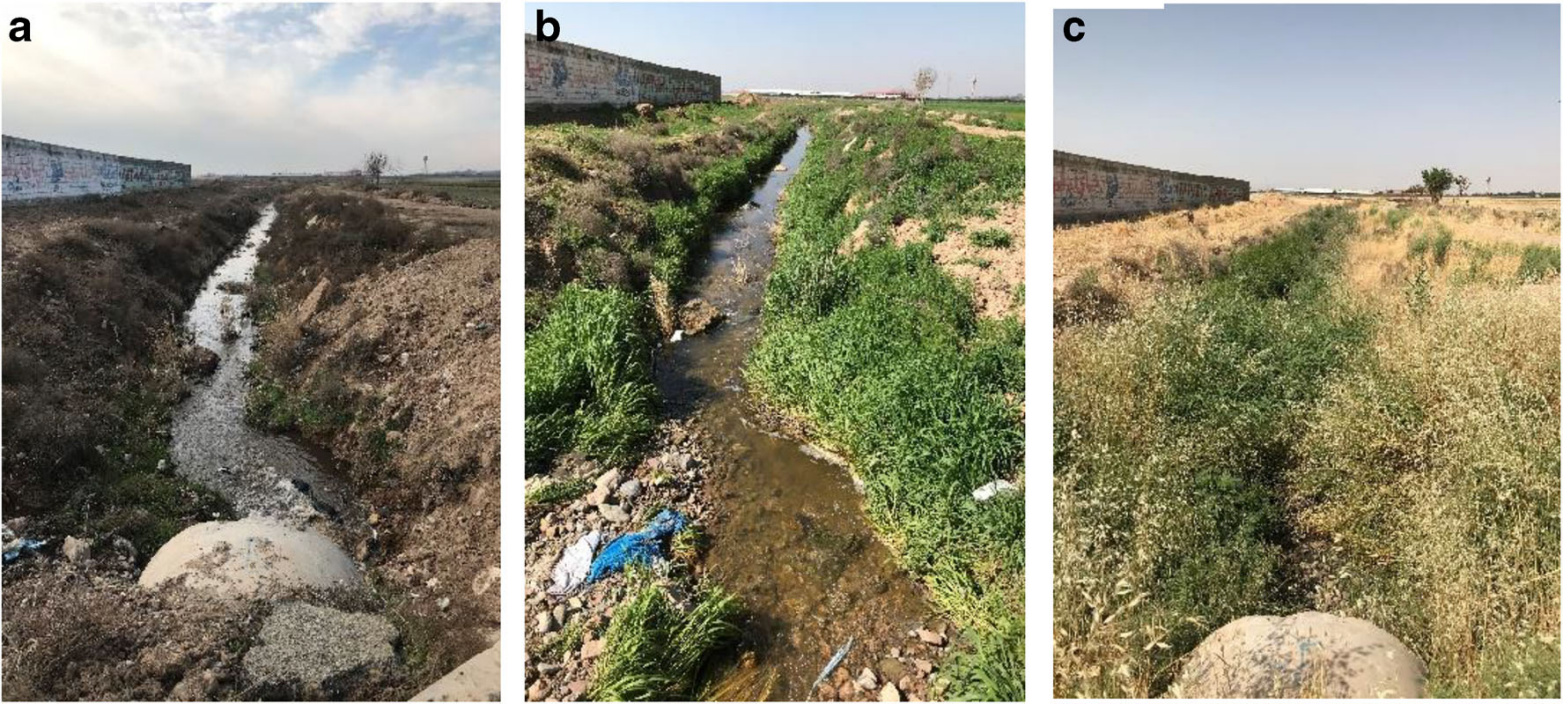
The former effluent outlet (channel) in winter (a), spring (b), and summer (c)

**Fig. 3:**
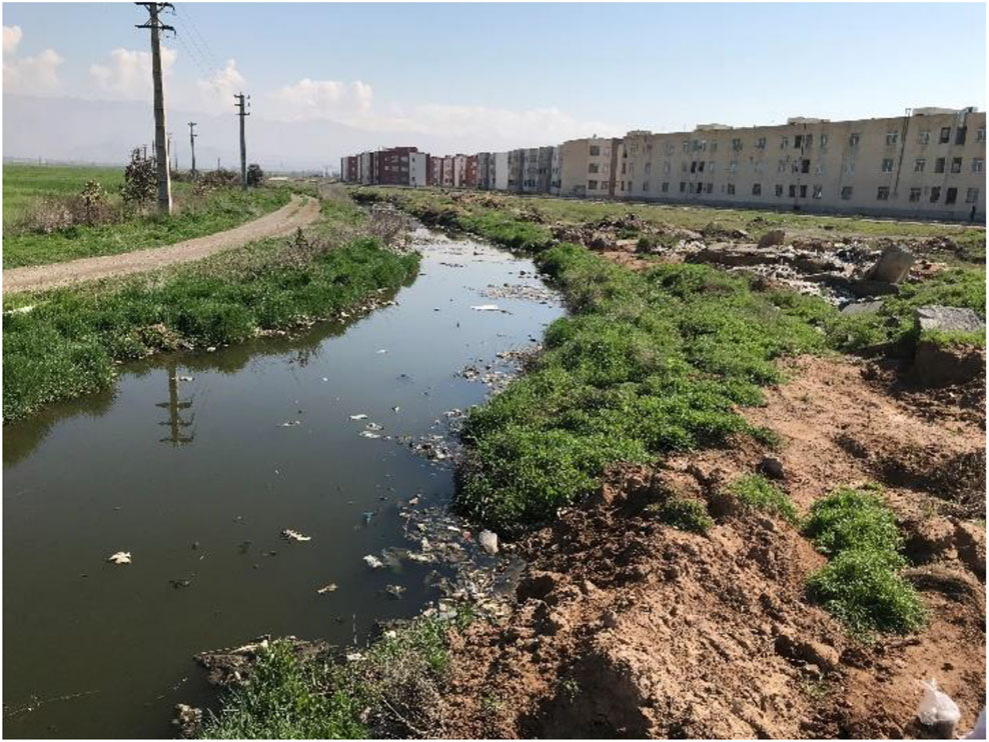
Proximity of residential area and effluent pond

Nazarabad municipal wastewater treatment plant is located in Nazarabad city at the west of Alborz province. This newly established treatment plant includes an extended aeration activated sludge system. A total amount of 30 liters of effluent from the treatment plant was sampled and transported to hydro-chemistry laboratory under standard conditions. Acidity (pH), electrical conductivity (EC), total dissolved solids (TDS), salinity (Sal), and temperature (T) were the five in situ parameters measured by pH meter and multi-parameter devices. The former effluent outlet (channel), with the specific UTM coordinates of 0461471 (longitudinal) and 3977448 (latitudinal), was located at an altitude of 1181 m. Figure 2 represents the former effluent outlet in three different seasons of winter, spring, and summer. The mentioned outlet is now closed due to the farmers’ dissatisfaction because the transferred wastewater through the channel was mainly used for irrigation purposes despite its polluting effect. For some time now, the treated wastewater has been released into a long pond near the treatment plant which is also close to the residential area (Fig. 3). Table 2 illustrates the characteristics of treated municipal wastewater sampled from inside the Nazarabad treatment plant before releasing into the pond.

**Table 2:** Characteristics of treated municipal wastewater sampled from inside Nazarabad treatment plant and recommended Iranian standard limits for artificial recharge of aquifer

	Parameter	Value	Method/device	Unit	Standard limit (mg/l)
Physico-chemical parameters	T	27	Multi-parameter and pH meter	Centigrade	_
	Sal	0.3	Multi-parameter	_	_
	EC	618	Multi-parameter	μS/cm	_
	TDS	308	Multi-parameter	mg/l	_
	pH	7.55	pH meter	_	5–9
Major ions (anions and cations)	Na^+^	109	ICP-MS	mg/l	_
	K^+^	12.41	ICP-MS	mg/l	_
	Ca^2+^	40.66	ICP-MS	mg/l	_
	Mg^2+^	25.72	ICP-MS	mg/l	100
	CO_3_^2−^	0.0	Titration	mg/l	_
	${\mathrm{HCO}}_3^{-}$	250.1	Titration	mg/l	_
	SO_4_^2−^	172.78	Turbidity meter	NTU	400
	Cl^−^	102.95	Argentometric	mg/l Cl^−^	600
Pollution indicators	BOD5	10	Respirometric	mg/l O_2_	30
	COD	35	Closed reflux titrimetric	mg/l O_2_	60
	NO_3_^−^	43	Brucine	mg/l NO_3_^−^	**10**
	PO_4_^3−^	1.55	Stannous chloride	mg/l PO_4_^3−^	6
	Total coliforms	2	Multiple tube technique	MPN/100 ml	1000
	Fecal coliforms	2	Multiple tube technique	MPN/100 ml	400

### Soil column design and filling

Column experiment recreates field conditions well and is appropriate to appraise the long-term release of chemical constituents from soil into the water bodies (Naka et al. 2016). Among the various materials used to make laboratory columns, three types, acrylic, glass, and stainless steel, are the most common. Originally, one plexiglass (acrylic) column with specified height of 45 cm and inner diameter of 5 cm was designed and built. Plexiglass columns do not react with soluble materials, have relatively good resistance, and are available in different diameters (Lewis and Sjöstrom 2010; Weihermüller et al. 2007). In order to monitor changes continuously, in addition to the end output of the column, three additional output holes with a diameter of 0.6 mm with regular and equal distances (15 cm) were embedded in the body of the column at 7.5, 22.5, and 37.5 cm. At the end and top of the column, two perforated plexiglass boards were installed. The end board was inseparable and enclosing, while the top board was detachable and placed on the surface of the soil. Perforated boards are used to prevent disturbing factors of sidewall flow (Corwin 2000; Sentenac et al. 2001) and fingering (Selker et al. 1999; Glass et al. 1989). For convenience, laboratory valves were installed at the top and bottom of the column to make it possible to control the entry and exit of effluent and thus to measure parameters such as hydraulic conductivity.

The entire column and its accessories were weighted before filling with the representative soil. To fill the plexiglass column using dry (or damp) packing method (Bégin et al. 2003; Communar et al. 2004), the soil was gradually poured into the column and compacted with a plastic rod (Oliviera et al. 1996). In this method, the height of the soil inside the column should be a maximum of 1 cm per embankment. This procedure is continued until the column is filled with compacted soil. Prior to filling, two layers of sterile gauze were placed at the end of the column (more precisely on the lower latticed plexiglass board) to prevent soil from spilling out of the end hole. The bulk density which was used for filling is 1.81 *gr*/*cm*^3^.

### Measuring parameters

Before starting the experiment, the *porosity (θ*_*PV*_*)* parameter was measured by using a cylindrical container with specified dimensions. This measurement was performed three times and finally its mean value was considered. In this way, first, the container was weighed once before and after filling with the soil, and then it was placed in a water pan to get soaked completely. Then, after re-weighing, the difference between the weight of dry and wet (saturated) soil was obtained and divided by the volume of the container. Before measuring the porosity, the soil must be fully compacted inside the chamber. The *bulk density* parameter was calculated by dividing the unsaturated weight (dry soil weight) by the volume of the cylindrical container (total volume). This parameter, like porosity, is averaged after several measurements (Table 1).

The *hydraulic conductivity (K)* parameter was measured for several times, and finally the average value of 252.73 cm/day was considered. In this study, the falling head permeability test was used to determine the hydraulic conductivity of soil because it was easier to measure in the laboratory. To do this, the treated wastewater that had previously been poured into a standpipe entered the column from the bottom by opening the embedded laboratory valve and exited from above and was poured into another container. The change in water height (hydraulic head) was recorded in the standpipe as well as the corresponding time, and by placing this information in the following formula, hydraulic conductivity was obtained (Delleur 1999):

1 $$ K=\frac{r^2L}{R^2t}\ln \frac{h_1}{h_2} $$

where *r*^2^ and *R*^2^ are the radii of the standpipe and of the specimen, respectively, *L* is the length of the specimen, and *h*_1_ and *h*_2_ are the heads at the beginning and at a later time *t*.

The above formula simplifies the following formula (Das 1985):

2 $$ K=2.303\ \frac{aL}{At}{\mathit{\log}}_{10}\frac{h_1}{h_2} $$

where *a* and *A* are the cross-sectional area of the standpipe and of the specimen, respectively.

### Soil column experiment

The laboratory setup includes a peristaltic pump, a treated wastewater reservoir which maintains a hydraulic constant head, a plexiglass column, and scaled beakers for collecting effluent samples. The experiment was performed using a peristaltic pump at the lowest flow rate of 3.075 ml/min to provide maximum time for soil and wastewater interactions. In this regard, the plan of 12 h wetting and 12 h drying cycle was selected. During this 6-day period experiment, constant pressure head and seepage face boundary conditions were established above and below the column, respectively.

Sampling of effluent from the column is based on *pore volume (PV)*, which is the product of the volume of the column (at the corresponding height) by soil porosity. In simpler terms, pore volume is equivalent to the volume of soil filled with water (Kirkham and Powers 1972). All the calculation results of pore volumes for each output hole are listed in Table 3. As mentioned earlier, there are 4 output holes in the column: 3 holes in the body (0.6 mm in diameter at regular intervals) and 1 hole at the end of the column. In order to sample each hole, the openings of the other three holes were closed. For example, when sampling the first hole at the top (having a volume equal to *PV*_1_ as shown in Table 3), the bottom three holes were closed so that the exchanged wastewater only came out of the open hole; or similarly, for sampling from the second hole, the openings of the other three holes were blocked so that only the second hole could be sampled. In this way, small samples of soil water were taken from the column at different depths. This was easier to do for the end hole because it had a laboratory valve. Also, the sampling order was as follows: in each series, the first, the second, the third, and the fourth hole were completely sampled, respectively, and then the next series of collecting sample began. Finally, sampling was completed in 20 series of sampling from each hole (80 samples from the column in total), and the output samples of all 5 pore volumes taken in succession from each hole were combined (e.g., PV1 to PV5 (PV1–5) of the first hole or PV10 to PV15 (PV10–15) of the third hole were mixed together). This means that, finally, 4 samples from each hole and a total of 16 samples from the column were analyzed for physico-chemical parameters, major ions (i.e., cations and anions), and trace elements. The findings will be described later in “Laboratory analysis of wastewater”. Approximately, 17 l of municipal wastewater exchanged from the soil column. At every stage of sampling, physico-chemical parameters such as EC (μS/cm), pH, TDS (mg/l), T (°C), and salinity were immediately measured on site using portable devices. Moreover, the trace element samples after acidification (pH < 2.0 by adding HNO3) were sent to the laboratory for inductively coupled plasma mass spectroscopy (ICP-MS) analysis, and other parameters including ${\mathrm{SO}}_4^{2-}$, ${\mathrm{HCO}}_3^{-}$, ${\mathrm{CO}}_3^{2-}$, Cl^−^, Ca^2+^, and Mg^2+^ were also measured. The principles of keeping samples in the laboratory were also fully observed; during the whole test, the samples taken were stored in the refrigerator at the standard temperature of 4 °C. At the end of the recharge experiment, the plexiglass column was cut into three equal 15 cm sections, and the soil in each section was investigated by ICP-MS analysis.

**Table 3:** Calculation of pore volumes assigned to each specific height of the soil column using measured porosity in the sedimentology laboratory

PV number	Height (cm)	Porosity (*θ*_*PV*_)	Column volume (= Πr^2^h)	PV value (ml)
PV1	0–7.5	0.38	147.18	55.93
PV2	7 .5–22.5	0.38	441.56	167.79
PV3	22.5–37.5	0.38	735.93	279.66
PV4	37.5–45	0.38	883.12	335.59
Total wastewater passing through column after each exchange				**838.97**
Total wastewater passing through column after 20 exchange				**16,779**

### HYDRUS-1D software

The basics of HYDRUS-1D goes back to the original work of van Genuchten (Šimůnek et al. 2012), but the main development of this software took place in 2008 with the release of version 4 (Šimůnek and van Genuchten 2008; Šimůnek et al. 2016). In this investigation, version 4.17.0140 of HYDRUS-1D was used to simulate the transfer of heavy metals in the soil (Radcliffe and Šimůnek 2010). This software can be downloaded from the pc-progress site at https://www.pc-progress.com/en/default.aspx.

The Richards equation is a basic equation of water movement in unsaturated soil, and its so-called “potential” form (1) is described as (Novák and Hlaváčiková 2019):

3 $$ c\left({h}_w\right)\frac{\partial {h}_w}{\partial t}=\frac{\partial }{\partial z}\left[k\left({h}_w\right)\frac{\partial {h}_w}{\partial z}\right]+\frac{\partial k\left({h}_w\right)}{\partial z}\pm S\left(z,t\right) $$

where c(*h*_*w*_) is specific soil-water capacity [*L*^−1^], *h*_*w*_ is the water pressure head [L], *t* is time [*T*], *k* is the unsaturated hydraulic conductivity function [*LT*^−1^], *z* is the spatial coordinate [L], and *S* is water outflow (negative sign) or water inflow (positive sign) from or to soil volume under consideration or in other words the sink term [*L*^3^*L*^−3^*T*^−1^ or *LT*^−1^].

In simulation models of one-dimensional water movement in homogeneous soils and under isothermal conditions, a modified form of the Richards Eq. (2) is often used which considers that the air phase plays an insignificant role in the liquid flow process and that water flow due to thermal gradients can be ignored. The governing one-dimensional uniform water flow equation is described as (Phogat et al. 2020; Šimůnek et al. 2013):

4 $$ \frac{\partial \theta \left({h}_w\right)}{\partial t}=\frac{\partial }{\partial z}\left[k\left({h}_w\right)\left(\frac{\partial {h}_w}{\partial z}+1\right)\right]-S(z) $$

where θ is the volumetric soil-water content [*L*^3^*L*^−3^ or −].

In this study, numerical modeling was done in three steps: firstly, plotting *breakthrough curve (BTC)*; secondly, parameter optimization by HYDRUS inverse modeling; and thirdly, prediction modeling. The obtained parameters of the first two steps were requisite to reach the final simulation (third step). In step one, after conducting a tracer test, BTC gives out the value for *longitudinal dispersivity (D*_*l*_*)*. This parameter is used to optimize and extract *adsorption isotherm coefficients (K*_*d*_ *and β)* in step two. In the last step, 5-year prediction modeling was performed in HYDRUS-1D by using mentioned optimized parameters.

## Results and discussions

### Laboratory analysis of wastewater

#### Physico-chemical parameters

**Fig. 4:**
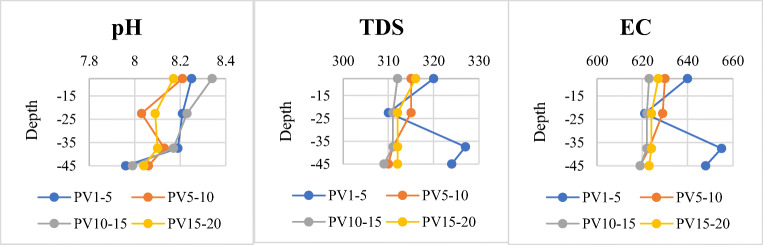
Changes in physico-chemical parameters of soil column effluent samples. PV1–5 refers to the 1st to 5th sampled pore volumes (PV1 to PV5) from the first (7.5 cm), second (22.5 cm), third (37.5 cm), and fourth (45 cm) outputs separately

In general, it can be understood from Fig. 4 that all physico-chemical parameters have unrelated trends with depth; however, their final value has increased compared to their initial value in the primary wastewater. As can be seen from comparing EC and TDS charts, their change curves are very similar. Neither of these two parameters has changed much, with the minimum and maximum values of 618 and 655 μS/cm for EC and 308 and 327 mg/l for TDS. Numbers of 618 and 308 belong to the pre-experiment wastewater. The pH of the incoming wastewater was 7.55, and at the end of the experiment, the pH value increased by 0.49 (8.04–7.55 = 0.49). The range of pH changes in the effluent samples was between 8.25 at the beginning and 8.04 at the end of the recharge, which indicates that the effluent becomes more acidic as it passes through the soil. Also, salinity of the input and all output wastewater samples showed a constant value of 0.3 throughout the soil column experiment, so the trend chart of this parameter has not been traced.

#### Major ions

**Fig. 5:**
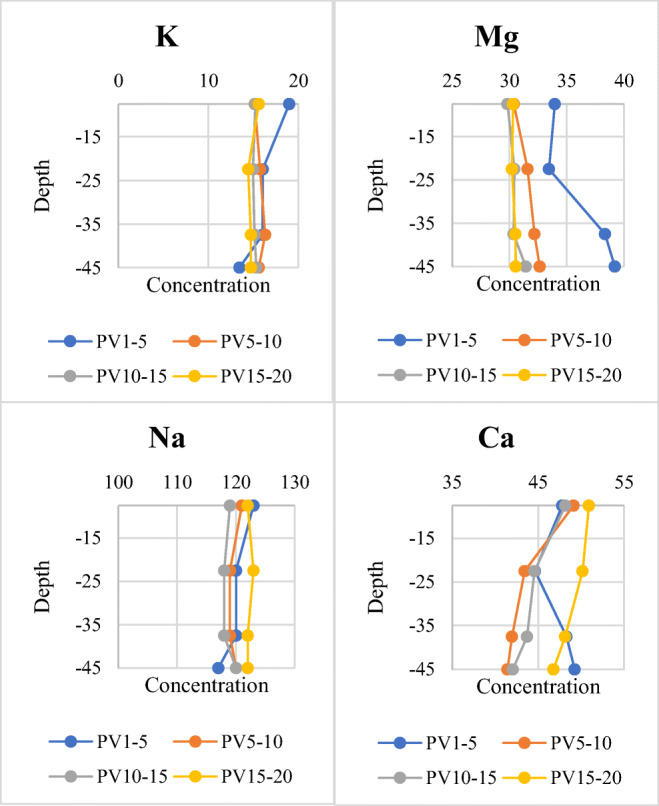
Changes in cation concentrations of soil column effluent samples. PV5–10 refers to the 5th to 10th sampled pore volumes (PV5 to PV10) from the first, second, third, and fourth outputs separately

In all soil water samples collected from output holes as explained in “Soil column experiment”, the concentration of the major cations (Na, K, Mg, and Ca) has increased compared to their concentrations in the primary wastewater. The concentration trends of these four cations are not very related to depth. Moreover, the range of concentration changes is low for each cation; for instance, during the entire test period, the difference between the highest and the lowest concentration for potassium ion (*K*^+^) was only 6.56 mg/l. This value was equal to 10.23, 13.47, and 14 mg/l for Ca, Mg, and Na, respectively. Of all these cations, Na had the highest concentration in effluent during the entire recharge experiment; hence it is the most abundant major cation in effluent (Fig. 5).

**Fig. 6:**
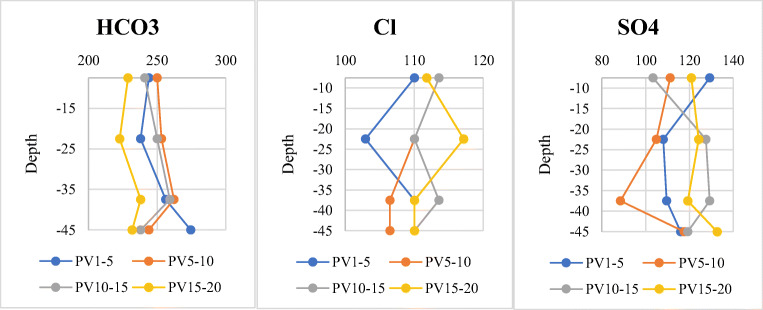
Changes in anion concentrations of soil column effluent samples. PV10–15 refers to the 10th to 15th sampled pore volumes (PV10 to PV15) from the first, second, third, and fourth outputs separately

In addition to cations, changes in the concentration of three major anions (${SO}_4^{2-}$, *Cl*^−^, and ${HCO}_3^{-}$) have also been analyzed in this experiment (Fig. 6). The concentration of sulfate and bicarbonate, unlike chloride, has decreased in the output samples compared to the primary effluent. Although ${HCO}_3^{-}$ shows a more regular trend of change, its change in concentration is irrelevant to depth, just similar to ${SO}_4^{2-}$ and *Cl*^−^. Needless to say that nitrate (${NO}_3^{-}$) was detected upper than the maximum permissible limit (43 mg/l ${NO}_3^{-}$) in the primary wastewater (Table 2). Nitrate can be transported from agricultural lands by both surface runoff and subsurface leaching because of the high mobility in the soil profile (Selim 2013) which seriously induces groundwater contamination (National Research Council 1984).

#### Trace elements (TEs)

**Fig. 7:**
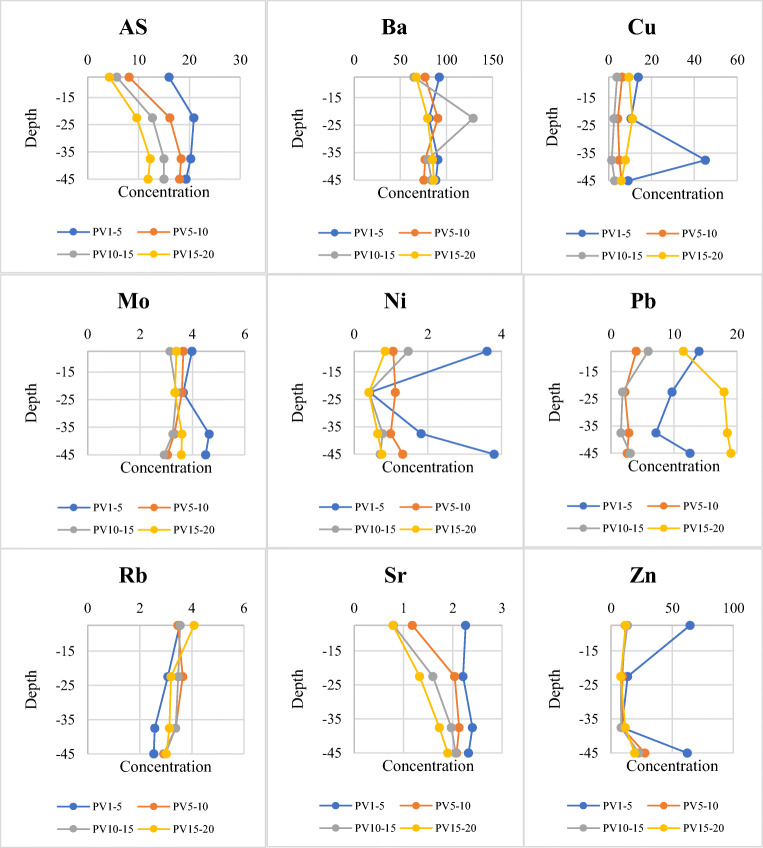
The changes in trace element concentrations of effluent samples while performing the experiment. PV15–20 refers to the 15th to 20th sampled pore volumes (PV15 to PV20) from the first, second, third, and fourth outputs separately

The 10 most important detected trace elements, hereafter referred to as TEs, in the pre- and post-experiment wastewater samples are As, Ba, Cr, Cu, Mo, Ni, Pb, Rb, Sr, and Zn. Cd and Tl have also been detected but their values were minor and under the detection limit. Table 4 depicts the results for ICP-MS analysis of TEs in primary wastewater and their suggested standard limits for aquifer recharge along with the permissible standard announced by the FAO and WHO (FAO 2003; WHO 2006). According to the table, all the TEs in the treated municipal wastewater sample from the Nazarabad treatment plant are below the recommended standard for groundwater recharge. Figure 7 illustrates the changes in TE concentrations in effluent samples while performing the experiment. It is observable that, with the exception of As and Sr, there is not a clear relationship between the TE concentration and depth of the soil column. Furthermore, only these two elements exhibit a regular decrease in concentration over time.

**Table 4:** ICP-MS analysis results of the trace elements in primary wastewater and their suggested standard limits for aquifer recharge *(*Iranian Department of Environment 2019*)* and irrigation *(*FAO 2003*;* WHO 2006*)*

Heavy metal	Value (ppb)	Standard limit	
		Department of Environment	FAO/WHO
As	7.55	100	100
Ba	30.56	1000	–
Cd	<1	100	10
Cr	9.8	1000–2000	100
Cu	2.24	1000	200
Mo	6.16	10	10
Ni	2.02	2000	200
Pb	<0.1	1000	5000
Rb	11.02	–	–
Sr	792	–	–
Tl	<0.1	–	–
Zn	4.47	2000	2000

### Laboratory analysis of soil

#### Trace elements (TEs)

On an ecological hazard basis, heavy metal/metalloid sequence in the soil is as follows: Se > Tl > Sb > Cd > Hg > Ni > Cu > Cr > As > B (Vodyanitskii 2013). Contrary to previous assumptions, it has been found that factors can enhance the mobility of these metals in the soil and, as a result, further leaching into groundwater (McBride 1995). Even complex pollution, caused by a variety of HMs, can exacerbate the contamination of individual heavy metals, for instance, Cu + Pb > Pb > Cu (Su et al. 2014). Soil is a natural filter, and its self-purification rate is a key factor in removing such metals and attenuating their migration rates to deeper parts of the soil (Keesstra et al. 2012; Plekhanova 2017). Otherwise, this can lead to health risks (Hussain and Qureshi 2020; Todd and Mays 2005).

**Fig. 8:**
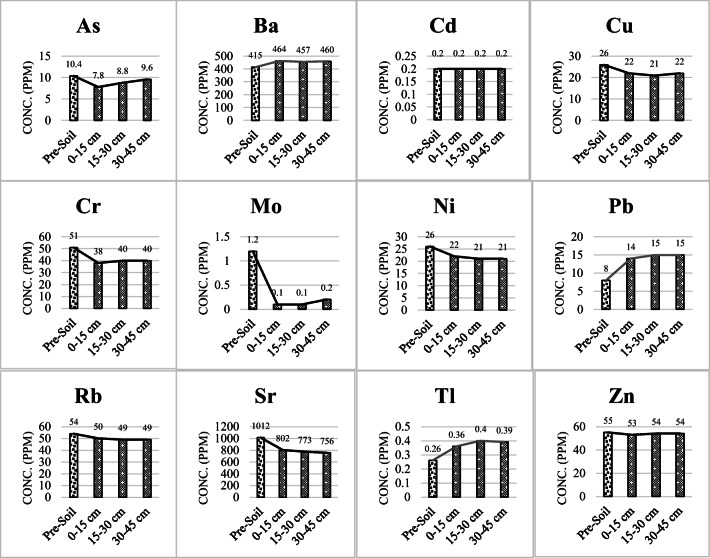
Changes in the concentration of HMs in the soil after the completion of recharge experiment and comparison with their initial amount

Table 5 represents the concentrations of pre-soil TEs compared to the Iranian standards for alkaline soil. Based on these standards, the representative soil sample taken from the farmlands irrigated with treated urban wastewater is not polluted; however, it is rich in Ba and Sr which releasing them into recharging water and then their further transportation through soil and water might be potentially hazardous for groundwater protection over an extended period of irrigation. As it was previously mentioned, the post-experiment soil of 0–15, 15–30, and 30–45 cm height were analyzed for TEs using ICP-MS analysis. Changes in the concentration of HMs in the soil in terms of height after the completion of recharge experiment and comparison with their initial amounts in the soil are shown in Fig. 8. In general, except Ba, Pb, and Tl, the concentration of all TEs in the soil has decreased after the experiment compared to the primary soil. Cd is the only heavy metal that does not show any concentration change in the soil due to its low concentration in the soil of the area and the wastewater passing through it. Regarding this metal and Tl, it can be said that, respectively, due to having a constant and very low concentration in the soil and the inability to extract the required coefficients, simulation of these metals has not been carried out.

**Table 5:** Concentrations of pre-soil trace elements compared to the Iranian standards for alkaline soil *(*Iranian Department of Environment 2019*)*

Heavy metal	Value (ppm)	Standard limit for soils with pH > 7		
		Groundwater recharge protection	Environmental conservation	Human (agriculture)
As	10.4	100	17	40
Ba	415	2000	500	600
Cd	0.2	20	3.9	5
Cr	51	100–3000	0.4–64	2–110
Cu	26	1500	63	200
Mo	1.2	100	4	40
Ni	26	600	50	110
Pb	8	300	300	75
Rb	54	–	–	–
Sr	1012	–	–	–
Tl	0.26	4	0.9	5
Zn	55	3000	200	500

### Software studies

#### Plotting *breakthrough curve (BTC)* to obtain longitudinal dispersivity (*D*_*l*_) by using inverse method in HYDRUS-1D

In simple and concise terms, a *BTC* represents the relationship between the concentration of a tracer, mostly a salt, and time (Kirkham and Powers 1972; Novák and Hlaváčiková 2019). In this study, we have used *potassium chloride* as the tracer; therefore the influent wastewater was switched with 1 l of KCl solution (5390 μS/cm). The EC of the solution leaching from the soil column was accurately measured in 20 volumes of 50 ml by the multi-parameter device. The duration of solution sampling was also recorded per 50 ml volume. In other words, 20 samples of 50 ml which was equivalent to the total volume of the input KCl solution were sampled at the end of the tracer test. The gained experimental values of EC were given to the model as input data to plot the BTC using inverse method of HYDRUS-1D. Finally, parameter of *longitudinal dispersivity (D*_*l*_*)* was obtained from the *BTC*: 14.81 m which is suitable for sandy soils. Achieving a reliable value for this parameter is fundamental because it is used in the subsequent modeling (Bromly et al. 2007). Clay content followed by column diameter is the most important factor controlling dispersivity (Bromly et al. 2007).

#### Obtaining *adsorption isotherm coefficients* of *K*_*d*_ and *β* for each metal separately using inverse method

In this step, as the previous one, the inverse method is used to obtain the required parameters. Table 6 demonstrates the obtained adsorption isotherm coefficients (*K*_*d*_ and *β*) for various TEs together with their standard deviation and confidence limits. To obtain these coefficients, the dispersivity parameter obtained from the previous step and the data related to the concentrations present in different depths of the soil after recharge experiment have been used (Radcliffe and Šimůnek 2010).

**Table 6:** Obtained adsorption isotherm coefficients (*K*_*d*_ and *β*) for various trace elements using inverse method of HYDRUS-1D along with their standard deviation and confidence limits

Element	Variable	Value	S. E. coeff.	95% confidence limits	
				Lower	Upper
As	K_d_ [*ml*/*g*]	1.86E+01	0.23923E+02	−0.28536E+03	0.32258E+03
	β [−]	1.34E+00	0.49702E+00	−0.49773E+01	0.76529E+01
Ba	K_d_ [*ml*/*g*]	1.24E+01	0.80356E+02	−0.10087E+04	0.10334E+04
	β [−]	1.17E+00	0.73696E+00	−0.81948E+01	0.10533E+02
Cr	K_d_ [*ml*/*g*]	1.08E+01	0.38076E+02	−0.47295E+03	0.49464E+03
	β [−]	1.23E+00	0.68699E+00	−0.75030E+01	0.99549E+01
Cu	K_d_ [*ml*/*g*]	9.26E+00	0.20608E+02	−0.25258E+03	0.27111E+03
	β [−]	1.48E+00	0.59941E+00	−0.61332E+01	0.90989E+01
Mo	K_d_ [*ml*/*g*]	1.09E+01	0.11781E+01	−0.40472E+01	0.25890E+02
	β [−]	6.30E−01	0.55672E−01	−0.77367E−01	0.13374E+01
Ni	K_d_ [*ml*/*g*]	1.00E+01	0.30812E+02	−0.38148E+03	0.40151E+03
	β [−]	1.48E+00	0.82833E+00	−0.90462E+01	0.12003E+02
Pb	K_d_ [*ml*/*g*]	1.43E+01	0.27675E+02	−0.33737E+03	−0.33737E+03
	β [−]	1.26E+00	0.60556E+00	−0.64339E+01	0.89546E+01
Rb	K_d_ [*ml*/*g*]	9.48E+00	0.39774E+02	−0.49588E+03	0.51485E+03
	β [−]	1.19E+00	0.70957E+00	−0.78263E+01	0.10205E+02
Sr	K_d_ [*ml*/*g*]	1.31E+01	0.81975E+02	−0.10284E+04	0.10547E+04
	β [−]	1.11E+00	0.82831E+00	−0.94176E+01	0.11631E+02
Zn	K_d_ [*ml*/*g*]	9.31E+00	0.34205E+02	−0.42529E+03	0.44392E+03
	β [−]	1.20E+00	0.66486E+00	−0.72471E+01	0.96484E+01

#### Five-year period modeling of the impact of heavy metals (HMs) in treated municipal wastewater on groundwater resources in Nazarabad plain

**Fig. 9:**
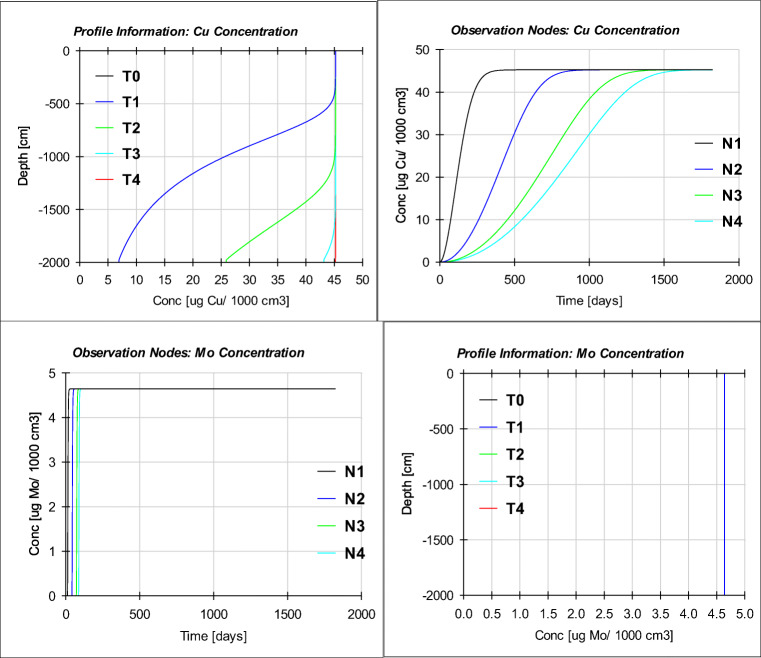
Five-year simulation of the impact of treated municipal wastewater on groundwater resources in Nazarabad plain. The simulation of TE concentration in the soil solution at different observation nodes N1, N2, N3, and N4 represented for the depth of 500 cm, 1000 cm, 1500 cm, and 2000 cm, respectively, and the different times T1, T2, T3, and T4 represented for 456, 912, 1368, and 1825 days, respectively

The third and final step of prediction modeling was performed by entering some important input data including soil depth of 20 m and a simulation duration of 5 years; also, the corresponding boundary conditions for the upper and lower boundaries were considered constant pressure head and free drainage, respectively. Figure 9 and Table 7 illustrate the final results for this simulation.

**Table 7:** Time of reaching equilibrium conditions (day) for different elements based on the prediction model results (numerical modeling)

Element	Time of reaching equilibrium conditions (day)
Mo	100
Cr	250
Rb	300
Zn	500
Ni	550
Ba	650
Sr	700
Pb	800
As	1300
Cu	1600

According to the results, three metals including molybdenum (Mo), chromium (Cr), and rubidium (Rb) have respectively the highest mobility and are balanced in a shorter time. In contrast, the other three metals involving copper (Cu), arsenic (As), and lead (Pb) have the least mobility (the most absorption by soil) and are slower to become balanced. The other heavy metals are somewhere between these two groups. The highest mobility indicates the lowest adsorption by the soil, and vice versa. Reaching the equilibrium rate for the rare elements during 1825 days (= 5 years) was as follows: Mo > Cr > Rb > Zn > Ni > Ba> Sr > Pb > As> Cu. In general, the diagrams show the leaching phenomenon that occurred at the beginning of the recharge; and sometime after the continuation of the recharge due to counterbalancing in the system, the concentration of ions in the effluent, and in other words the dissolution rate, remained constant until the end of 5 years. By increasing the time period of the model (more than 1825 days), it is possible to reduce the dissolution of metals after a period of constant stability and balance of the system.

The result of the 5-year prediction modeling shows a good correlation with real conditions; this means that just as molybdenum is highly mobile in nature, or that lead, copper, and other metals are less mobile, modeling results have also shown the same. Low mobility and high mobility metals in nature are among the low mobility and high mobility metals in the simulation results, respectively. The numbers for the correlations for some of the elements are as follows: Mo (*R*^2^= 0.99677, *RMSE* = 0.1257E+00), Sr (*R*^2^= 0.72938, *RMSE* = 0.4323E+01), Cu (*R*^2^= 0.71535, *RMSE* = 0.1287E+01), and As (*R*^2^= 0.60524, *RMSE* = 0.4620E+00).

In general, there are six factors affecting TE mobility and transport: soil pH, chemical speciation, soil organic matter, fertilizers and soil amendments, redox potential, and finally clay content and soil structure. Among these, pH is the main variable controlling the solubility, mobility, and transport of TEs because it influences many soil processes including TE sorption; in fact, sorption is pH dependent and increases as pH increases. In sandy soils, such as the soil of this study, ion sorption increases by 2–10 fold per one unit increase in pH (Carrillo-González et al. 2006). Under normal soil pH conditions, if the organic matter decomposes and forms a complex with HMs, the concentration of HMs in the soil solution increases. Thus, mobility and thereafter leaching rates increase; as a result, infiltration time is reduced. In contrast, the formation and accumulation of organic matter can retain HMs and reduce leaching rate as well as mobility through the soil-subsurface system (Berkowitz et al. 2014; Chu et al. 2014). As shown in Fig. 9, the dissolution of TEs after the start of irrigation with municipal wastewater has an increasing trend, and after several days of its onset (which varies for different elements), the dissolution rate has reached equilibrium; and this trend has continued until the end of 5 years. Based on Table 7, Cu was balanced in soil 16 times slower than Mo, and because Cu is less mobile and more likely to be trapped in the upper soil layer, it would have the potential to contaminate the topsoil, while Mo and other more mobile TEs showed a higher potential to contaminate groundwater.

Unlike most heavy metals, Mo is the most active in alkaline condition (pH > 6.5) (Alloway 2013; Hettiarachchi and Gupta 2007), and even, based on previously conducted batch and soil experiments (Wenguang and Selim 2020), at the neutral condition, its mobility is higher than that at the acidic condition. The results of the numerical modeling also revealed that due to the fact that the soil of the region is alkaline, this metal is the most active of the 10 simulated metals. In addition, the behavior of Mo in soil is more similar to that of sulfate than other metals (Alloway 2013), and since the amount of both Mo and ${SO}_4^{2-}$ after passing through the soil is lower than the initial value, it can be concluded that experimental modeling of this metal is also correct.

In the case of Cr, the second most mobile metal, the deduction from former batch experiments has shown that this metal is not easily absorbed by the soil and is highly sensitive to pH value. At a very acidic pH, chromium adsorption capacity is maximal (Zhang et al. 2018). This proves the result of the carried-out simulation with respect to Cr.

Based on the results of numerical modeling, Cu and Pb are among the metals with the least mobility (maximum absorption) in the soil. In confirming this conclusion, it can be explained that Cu has a lower solubility at high pH; thus copper deposits can form in alkaline soils (Alloway 2013). In addition, at pH greater than 4.61, the amount of Pb absorbed by the soil typically increases, so that in alkaline soils Pb absorption is high (Fetter et al. 2018). The capacity of clay minerals to adsorb Pb also increases at higher pH (Fetter et al. 2018), and due to the fact that these minerals are present in the soil of the research site, it is possible that the local soil increases the absorption of this metal. Besides, during the adsorption mechanism, arsenic (As) is preserved in soil, so its mobility is limited. As a matter of fact, when the soil pH is between 4 and 8, arsenic is least mobile. The presence of adsorbent clay minerals and soil pH are two important factors in determining the amount of As adsorption (Hassan 2018; Sherameti and Varma 2015).

Furthermore, according to the results of numerical modeling, Ba and Ni have moderate mobility compared to the other metals and are in the middle range. To justify this, it can be said that the solubility of Ni is highly dependent on pH, and in alkaline pH, Ni is present as hydroxide with limited solubility (Alloway 2013; Chotpantarat et al. 2011). Also, Ba is not very mobile in most soils, and even absorbing this metal from alkaline soils by plants is much more difficult than in acidic soils. In addition, in soils with high amounts of clay minerals and calcium carbonate (calcite), the mobility of barium is limited by surface adsorption and deposition. However, in the presence of chlorine ions, Ba is more active, and this can prevent the maximum mobility of this element in the soil (Alloway 2013) (experimental modeling has shown an increase in the concentration of chlorine anion compared to the initial value).

Zinc has shown relatively higher mobility (less absorption) than six metals including Ni, Ba, Sr, Pb, As, and Cu. In justifying this result, it can be said that the adsorption of Zn decreases in the alkaline environment and most probably in the presence of calcium carbonate (Jennie 1968). In general, the concentration of Zn per unit of pH decreases fivefold in the soil (Alloway 2013). Formerly conducted column tests specified that the obtained results from this method appropriately approximate the sorption behavior of zinc during its transport through the soil comparing to batch experiments (Behroozi et al. 2020).

Other simulated elements also showed good adaptation to their natural soil conditions. Regarding strontium (Sr), which its mode of sorption to the soil is pH dependent, multilayer adsorption occurs above pH 4.5 (Guillén 2018). Rubidium (Rb) is a radioactive element which tends to have a degree of mobility in soil (Schulz 1996). Its uptake in plants is strongly dependent on soil pH (Gjengedal and Steinnes 1994); the higher the soil acidity, the greater the uptake of Rb by vascular plants and fungi (Tyler 1983).

## Conclusions and recommendations

ICP-MS analysis of primary wastewater showed that all TE concentrations in effluent are within the recommended limits for aquifer recharge. Experimental modeling revealed that, with the exception of As and Sr, there is no regular decreasing or increasing relationship between the concentrations of the elements and depth of the soil column. Based on Iranian standards for alkaline soils, the primary soil was not contaminated; however, it was rich in Ba and Sr. Similarly, soil samples did not contain any contaminants after the end of the soil column experiment. In general, except Ba, Pb, and Tl, the concentration of all TEs in the soil has decreased after the experiment compared to the primary soil. According to the numerical simulation with HYDRUS-1D, copper (Cu), arsenic (As), and lead (Pb) have the most absorption by soil and are slower to become balanced. Molybdenum (Mo) has the highest mobility and is balanced in a shorter time.

The results of physical and numerical modeling show that the soil is somewhat capable of self-purification of heavy metals; while, due to the high hydraulic conductivity, the possibility of contamination of the aquifer with pollutants is high, the infiltration water will not reduce the quality of groundwater because of the low concentration of heavy metals. In fact, as long as the treatment plant works well, it does not pose a risk of groundwater pollution. Therefore, continuous monitoring of the characteristics of the treated effluent of the treatment plant is vital.

Due to the use of treated wastewater of Nazarabad municipal treatment plant in irrigation of farms and agricultural activities, it is recommended to monitor other unknown characteristics of wastewater, such as microbiological parameters, to finally ensure the consumption of effluent in this sector. It should be mentioned that previous reports provided by the refinery itself have indicated the presence of two different classes of helminths (*Taenia saginata* and *Ascaris lumbricoides*) in the treated wastewater. Besides, it is indispensable for the treatment plant officials to take the necessary measures to reduce the nitrate pollution in the effluent.

## Data Availability

All data are provided in the manuscript.
